# The Silent Culprit: Drug-Induced Fever Associated With Trimethoprim-Sulfamethoxazole

**DOI:** 10.7759/cureus.96994

**Published:** 2025-11-16

**Authors:** Maria Lages, Cristina Coelho, Susana Cadinha, Joana Barradas Lopes

**Affiliations:** 1 Allergy and Clinical Immunology, Unidade Local de Saúde do Algarve, Faro, PRT; 2 Allergy and Clinical Immunology, Unidade Local de Saúde de Gaia e Espinho, Vila Nova de Gaia, PRT

**Keywords:** diagnostic challenges, drug-induced fever, drug provocation test, hypersensitivity reaction, trimethoprim-sulfamethoxazole

## Abstract

Drug-induced fever (DIF) is often misdiagnosed, particularly when it occurs alongside an infection. Its hallmark is the resolution of fever upon drug withdrawal and rapid recurrence upon re-exposure. Although five potential mechanisms have been proposed to explain this phenomenon, they remain poorly understood, making DIF a challenging diagnosis. We report a case of trimethoprim-sulfamethoxazole-induced drug fever, demonstrated by reproducible symptom onset following a drug provocation test, with supportive evidence from positive intradermal and lymphocyte transformation tests. The diagnosis of DIF requires a high degree of suspicion and the thorough exclusion of other causes of fever, such as infections or inflammatory conditions. Increased awareness and understanding of DIF are essential to improve diagnostic accuracy and patient outcomes.

## Introduction

Drug-induced fever (DIF) is defined as a febrile response (T > 38 °C) that arises during pharmacological treatment and subsides after discontinuation of the suspected drug. Its onset is highly variable among drug classes and the underlying mechanism, but it most commonly appears after 7-10 days of drug administration and usually resolves within 48 hours of discontinuation [1,2]. The diagnosis requires the exclusion of alternative causes through a detailed medical history, physical examination, and appropriate laboratory investigation. Rechallenge with the suspected drug often results in a rapid recurrence of fever, typically within a few hours, which can help confirm the diagnosis [2,3]. According to Patel et al., five mechanisms have been identified to explain DIF, which can be classified as altered thermoregulatory control, administration-related reactions, direct pharmacologic effects, idiosyncratic responses, and hypersensitivity reactions [1]. This diagnosis remains challenging and requires a high index of clinical suspicion. The literature on this topic is limited, and the true incidence remains unknown due to frequent misdiagnosis and underreporting. Available data suggests that DIF may account for approximately 3-5% of all adverse drug reactions, but this likely underestimates the real incidence [1,4]. Raising awareness of this underrecognized clinical entity is essential to prevent unnecessary diagnostic procedures, inappropriate antibiotic use, and prolonged hospitalizations. We report a case of DIF associated with trimethoprim-sulfamethoxazole (TMP-SMX).

## Case presentation

**Figure 1:**
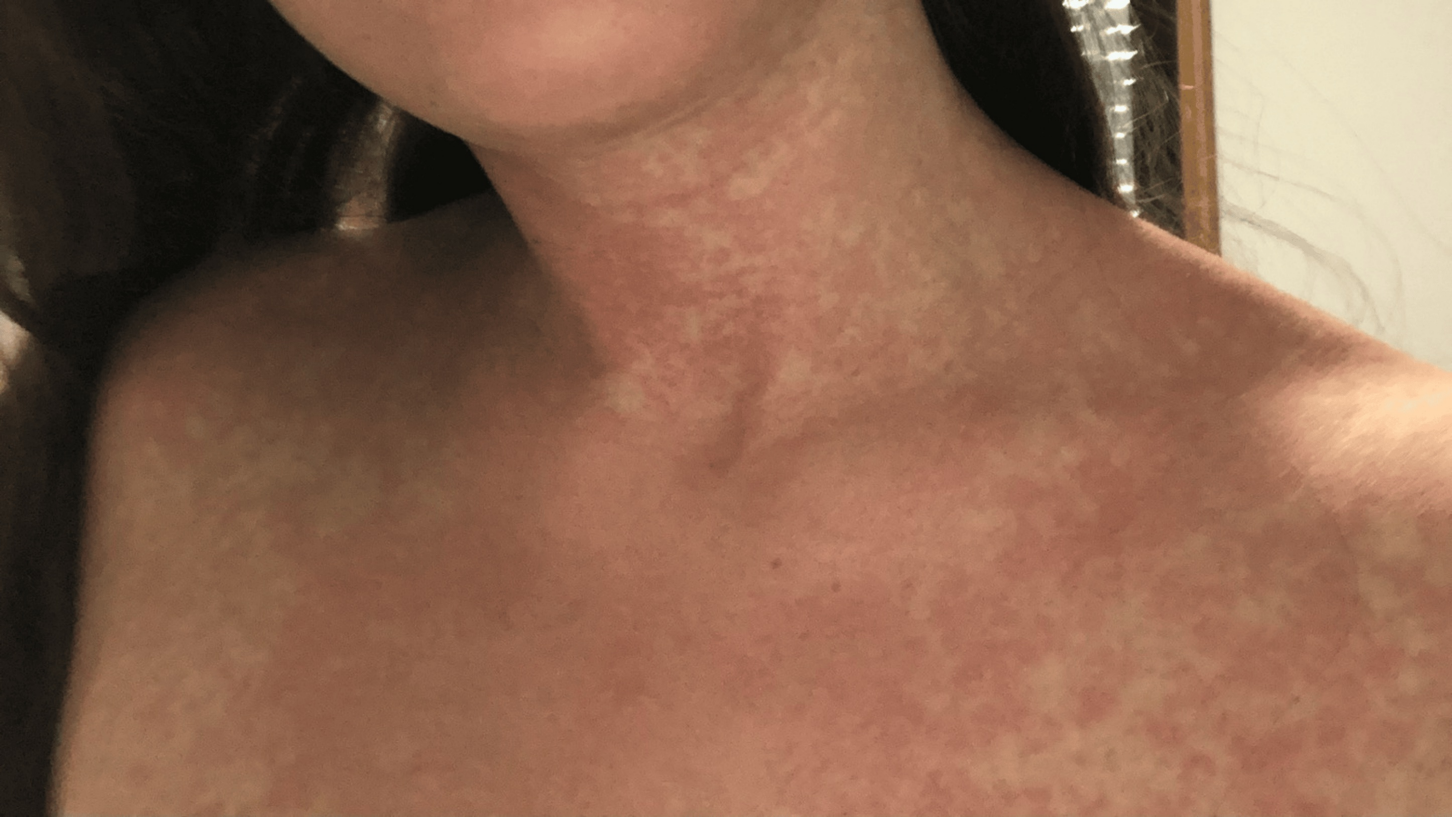
Macular rash on the trunk and upper limbs after the final dose on the eighth day of treatment with trimethoprim-sulfamethoxazole, characterized by multiple well-defined erythematous macules with pruritus.

A 21-year-old female patient with a history of recurrent urinary tract infections (UTIs) was referred to the Allergy and Clinical Immunology Department for suspected hypersensitivity to TMP-SMX. On the sixth day of TMP-SMX treatment for a UTI, she developed fever (maximum temperature of 39.9 °C), diarrhea, and myalgia. She subsequently self-medicated with ibuprofen 600 mg and paracetamol 1000 mg. After completing an eight-day course of TMP-SMX, she noted the appearance of a pruritic macular rash (Figure 1), prompting evaluation in the Emergency Department (ED). Laboratory analysis revealed relative lymphopenia, without leukocytosis or eosinophilia, and a C-reactive protein (CRP) level of 16.64 mg/dL (reference range: 0-0.5 mg/dL). Urinalysis was normal, with negative results in both blood and urine cultures. Following discharge with bilastine and prednisolone, the patient experienced complete resolution of the skin lesions within 2-3 days, and the fever subsided within 48 hours. As part of the initial allergy workup, patch testing with TMP-SMX 10% in petrolatum (Chemotechnique Diagnostics®) was performed, with negative results at 48 and 72 hours. Single-blind, placebo-controlled oral drug provocation tests (DPTs) with paracetamol (cumulative dose of 1000 mg) and ibuprofen (cumulative dose of 600 mg) were subsequently conducted, both yielding negative results. Eleven months after the initial reaction, a single-blind, placebo-controlled DPT with TMP-SMX was then performed, reaching a cumulative dose of 800 mg + 160 mg, administered in two divided doses 30 minutes apart [5]. Approximately four hours after the DPT, the patient developed fever (maximum temperature of 39.3 °C), which was unresponsive to paracetamol, and bradycardia. Additionally, erythema was observed on the cervical region, face, and palms, along with conjunctival hyperemia. The patient also reported frontal headache and cervical pain, with limited neck flexion. She was evaluated in the Allergy and Clinical Immunology Department and treated with paracetamol 1000 mg and ebastine 20 mg, leading to resolution of fever and initial improvement in cervical symptoms. However, a few hours later, while still under observation, worsening cervical pain and stiffness prompted referral to the ED, where laboratory tests revealed relative lymphopenia without leukocytosis or eosinophilia, and a CRP level of 11.34 mg/dL (reference range: 0-0.5 mg/dL). Urinalysis was normal, and blood and urine cultures were negative. A neurological assessment was performed due to suspected infectious or aseptic meningitis, prompting a lumbar puncture. Cerebrospinal fluid (CSF) analysis revealed no pleocytosis, protein abnormalities, or hypoglycorrhachia, excluding meningitis. Clinical improvement and resolution of fever occurred within 48 hours in the context of symptomatic treatment with paracetamol. The similarity to the initial episode reinforced the suspicion of DIF. Three months after the DPT, the lymphocyte transformation test (LTT) with TMP-SMX demonstrated probable sensitization, defined as a stimulation index (SI) ≥ 3 with at least two positive replicates out of 24 at any tested concentration. The results are presented in Table 1. Subsequently, skin prick testing with TMP-SMX (96 mg/mL) was negative, and intradermal testing (IDT) with a 1:100 dilution was positive on immediate and 48-hour readings. Based on these findings, avoidance of TMP-SMX was recommended. 

**Table 1:** Lymphocyte transformation test results with trimethoprim-sulfamethoxazole. TMP-SMX: Trimethoprim-Sulfamethoxazole; SI: Stimulation Index.

TMP-SMX (µg/mL)	SI	Positive replicates out of 24
0.1	20.4	14/24
1	21.2	14/24
10	7.5	7/24
100	<2	0/24

## Discussion

TMP-SMX is frequently prescribed due to its cost-effectiveness and broad-spectrum activity against common infections, including UTI, traveler's diarrhea, and skin and soft tissue infections caused by methicillin-resistant *Staphylococcus aureus*. Despite its clinical utility, TMP-SMX is associated with a range of adverse reactions, including sepsis-like syndrome and drug-induced aseptic meningitis [6,7]. In this case, the diagnostic approach required careful exclusion of infectious and inflammatory conditions. Infection was ruled out by the absence of a clinical focus, negative microbiological tests, and spontaneous resolution without antibiotic therapy. Aseptic meningitis was also excluded based on normal CSF parameters. CSF findings typically associated with TMP-SMX-induced meningitis include neutrophilic pleocytosis, mild protein elevation, normal glucose, and a negative gram stain [6], none of which were present in our patient. A sepsis-like syndrome was considered unlikely given the absence of organ dysfunction and systemic inflammatory response. Additionally, the patient was not on other medications commonly implicated in DIF, such as anticonvulsants, and had no evidence of idiosyncratic response or underlying disorders affecting thermoregulation. DIF can present a diagnostic challenge, particularly when occurring during antibiotic therapy, as it may be mistaken for infection recurrence. Recognizing this distinction is essential, as misdiagnosis can lead to the unwarranted continuation or escalation of antibiotics, with implications for antibiotic resistance. Considering DIF in the differential diagnosis of fever supports more appropriate antibiotic use and better patient outcomes. In this context, a high index of suspicion is essential to avoid unnecessary diagnostic workup, antibiotics overuse, and prolonged hospitalization. Fever as the sole or predominant manifestation is rare, observed in only 3-5% of reported drug fever cases [1,2]. Cutaneous signs are also uncommon, reported in approximately 18-29% of cases with DIF [1,8]. In our patient, both were present. Bradycardia, an atypical finding in febrile illnesses due to the usual pulse-temperature correlation (Liebermeister’s rule), is frequently reported in DIF and was documented in this case [9]. Laboratory features such as eosinophilia and leukopenia are variably reported [9-12]. In a case series of antibiotic-induced drug fever, CRP elevation was observed in 10 of 11 cases, with a median of 5 mg/dL (range: 3.2-22.9) [9,13,14]. This pattern was also observed in our case, with a significant increase in CRP levels during the febrile episode. The cutaneous manifestations observed during the febrile episode initially suggested a non-immediate hypersensitivity reaction, and the fever was interpreted in the context of a probable underlying infection. Consequently, the diagnostic approach included patch testing followed by a DPT. The diagnostic value of patch testing is limited, as it may lack sufficient sensitivity and can yield false-negative results [15,16]. However, the recurrence of fever approximately four hours after the DPT with TMP-SMX, in the absence of infection or other identifiable causes, raised the suspicion of a different mechanism. The allergy workup left some uncertainty regarding the underlying immunological mechanism. The patient had a positive DPT earlier than the timing of the initial reaction, within less than six hours, which may be compatible with either type I (IgE-mediated) or type IV (T cell-mediated) hypersensitivity. The immediate reading of the IDT was positive and remained positive at 48 hours. Given some concerns about possible irritant effects in the immediate reading of IDT with this drug at the recommended concentrations, it is difficult to definitively determine the mechanism involved. TMP-SMX is commonly reported to cause hypersensitivity reactions, which can involve the bioactivation of sulfamethoxazole into reactive metabolites, with susceptibility further influenced by genetic, metabolic, and immunological factors [15,17]. This interpretation aligns with current literature, which identifies hypersensitivity reactions as the most common mechanism of DIF [1]. This case highlights the diagnostic challenges posed by DIF and reinforces the importance of a comprehensive diagnostic approach [18-20]. A structured evaluation enabled the identification of an uncommon clinical presentation and clarification of the underlying immunological mechanism. The diagnosis was supported by clinical history, reproducibility of symptoms upon re-exposure, and positive IDT and LTT, confirming hypersensitivity to TMP-SMX and justifying its avoidance. Despite advances in the understanding of DIF, several gaps remain: there is no consensus on its definition, tools to estimate the probability prior to drug discontinuation are lacking, and the safety of rechallenge as the diagnostic gold standard is insufficiently studied. Large population studies are needed to confirm its safety, and vigilance should be reinforced given the increasing diversity of drugs implicated [9]. 

## Conclusions

DIF remains an underdiagnosed and often overlooked condition, particularly during antibiotic therapy, where it may be mistaken for infection recurrence. Diagnosis relies on a high index of suspicion, temporal association with drug exposure, resolution after withdrawal, and careful exclusion of alternative causes. Greater clinical awareness and diagnostic accuracy are crucial to ensure appropriate management and avoid unnecessary interventions. Moreover, the development of standardized diagnostic approaches could facilitate earlier recognition and support more effective patient care.
